# A Phylometagenomic Exploration of Oceanic Alphaproteobacteria Reveals Mitochondrial Relatives Unrelated to the SAR11 Clade

**DOI:** 10.1371/journal.pone.0024457

**Published:** 2011-09-14

**Authors:** Björn Brindefalk, Thijs J. G. Ettema, Johan Viklund, Mikael Thollesson, Siv G. E. Andersson

**Affiliations:** Department of Molecular Evolution, Evolutionary Biology Center, Science for Life Laboratory, Uppsala, Sweden; University of Wyoming, United States of America

## Abstract

**Background:**

According to the endosymbiont hypothesis, the mitochondrial system for aerobic respiration was derived from an ancestral Alphaproteobacterium. Phylogenetic studies indicate that the mitochondrial ancestor is most closely related to the Rickettsiales. Recently, it was suggested that *Candidatus* Pelagibacter ubique, a member of the SAR11 clade that is highly abundant in the oceans, is a sister taxon to the mitochondrial-Rickettsiales clade. The availability of ocean metagenome data substantially increases the sampling of Alphaproteobacteria inhabiting the oxygen-containing waters of the oceans that likely resemble the originating environment of mitochondria.

**Methodology/Principal Findings:**

We present a phylogenetic study of the origin of mitochondria that incorporates metagenome data from the Global Ocean Sampling (GOS) expedition. We identify mitochondrially related sequences in the GOS dataset that represent a rare group of Alphaproteobacteria, designated OMAC (Oceanic Mitochondria Affiliated Clade) as the closest free-living relatives to mitochondria in the oceans. In addition, our analyses reject the hypothesis that the mitochondrial system for aerobic respiration is affiliated with that of the SAR11 clade.

**Conclusions/Significance:**

Our results allude to the existence of an alphaproteobacterial clade in the oxygen-rich surface waters of the oceans that represents the closest free-living relative to mitochondria identified thus far. In addition, our findings underscore the importance of expanding the taxonomic diversity in phylogenetic analyses beyond that represented by cultivated bacteria to study the origin of mitochondria.

## Introduction

Mitochondria are eukaryotic organelles for aerobic respiration, thought to have originated sometime after the rise of oceanic and atmospheric oxygen levels roughly about 2.0×10^9^ years ago [1], [2]. Early phylogenetic analyses of ribosomal RNA and of components in the respiratory chain complexes suggested that the mitochondrion was derived from an Alphaproteobacterial endosymbiont [3], [4]. Consistently, comparative genomics analyses have identified aerobic respiration as ancestrally present in both the Alphaproteobacteria [5] and the proto-mitochondrion [6]. However, despite extensive debate, there is no consensus about the identities and characteristics of the partners involved in the endosymbiotic association.

On the host-side, the debate has focused on the extent to which the origin of mitochondria coincides with the emergence of eukaryotes. Several mutually incompatible models have been proposed. One suggests that the host was a relatively complex eukaryote, as explicitly stated in the Archezoa hypothesis [7]. A counter-argument raised against this model is that ancestrally amitochondriate eukaryotes have never been found [8]. Other models, such as the Hydrogen hypothesis, favour an archaeal methanogenic host that evolved into a eukaryotic cell upon the acquisition of mitochondria [9].

On the endosymbiont side, discussions have centred on the metabolic capability transferred to the host and the phylogenetic placement of the lineage from which the mitochondrial endosymbiont emerged. Since subunits of key enzymes of the respiratory chain complexes are encoded by all mitochondrial genomes and homologs are present in most of the sequenced alphaproteobacterial genomes, attempts to elucidate the origin of mitochondria have mostly focused on the system for aerobic respiration [10]–[12]. A few ribosomal proteins are encoded by some mitochondrial genomes and these have also been used to trace the mitochondrial ancestry [13], [14].

With the number of complete genome sequences from alphaproteobacterial species steadily growing, several recent studies have re-examined the placement of mitochondria in relation to contemporary alphaproteobacterial species [13]–[15]. Phylogenetic analyses of concatenated protein alignments have suggested that mitochondria are affiliated with the Rickettsiales [14], [15], an obligate intracellular clade with members that are well adapted to the cytosol of both metazoa [16] and protozoa [17]. However, phylogenetic analyses of single proteins have indicated different placements of mitochondria in relation to the Rickettsiales, as seen for example in a study of the mitochondrial phylome of *Reclinomonas americana* [13]. Of the single proteins analyzed, some indicated a placement of the mitochondria outside the Alphaproteobacteria, others as a sister-group to the Rickettsiales and the remaining suggested that they diverged within or represents a sister-group to the clade consisting of Rhizobiales, Rhodobacterales, Rhodospirilalles, Caulobacterales and Sphingomonadales [13].

A possible underlying reason for the difficulty in determining the specific placement of mitochondria in the alphaproteobacterial tree might involve unbalanced taxon sampling, as the current collection of completely sequenced genomes, including those of Alphaproteobacteria, is heavily biased towards medically and agriculturally relevant species. As such, the current sampling does not extensively cover the taxonomic diversity of species that inhabit environments in which mitochondria might have originated, such as the oxygen-producing marine photic zone. Given that Alphaproteobacteria are extremely common in these habitats with members of the SAR11 clade representing 30–40% of total cell counts in the oceans, it is of interest to examine the relationship between mitochondria and oceanic Alphaproteobacteria.

The SAR11 group of bacteria plays an important role in the oceanic carbon cycle. These bacteria have small cell volumes and grow slowly. The genome of *Candidatus* Pelagibacter ubique has recently been sequenced [18] and it currently is the only published genome from a species belonging to the SAR11 clade. The *Ca.* Pelagibacter ubique genome is 1.3 Mb in size and, with an average intergenic space of only 3 basepairs, it is one of the most compact of all bacterial genomes sequenced to date [18]. Interestingly, the first and only phylogeny inferred so far from a concatenated protein alignment that includes *Ca.* Pelagibacter ubique along with mitochondria and 71 other alphaproteobacterial species has identified *Ca.* Pelagibacter ubique as a sister-species to the clade encompassing mitochondria and the Rickettsiales [15].

In light of the points discussed above, a better sampling of oceanic bacteria might help to determine the nature of the mitochondrial progenitor. The largest marine metagenomic sequencing initiative performed to date, the Global Ocean Survey (GOS) [19] uncovered more than 6 million genes from the ocean surface waters, of which at least one third, perhaps up to as much as half, can be attributed to alphaproteobacterial species. Based on analysis of ribosomal RNA abundance, SAR11 is by far the most abundant alphaproteobacterial clade in this dataset, while other alphaproteobacterial orders such as SAR116 and Rhodobacterales (which includes the Roseobacter clade) are also well represented in the oceanic surface waters [20]. A clustering analysis of proteorhodopsin sequences affiliated with *Ca.* Pelagibacter ubique indicated extensive genetic diversity within this lineage [19]. Thus, the GOS data set provides the most comprehensive and diverse collection of SAR11 sequences to date.

The aim of this study was to examine the evolutionary relationship of aerobic respiration in mitochondria in relation to the homologous systems in oceanic bacteria. We have used a phylogenomic framework to select suitable marker genes for inferring the phylogenetic relationship of Alphaproteobacteria and mitochondria, and to extract an include alphaproteobacterial orthologs from the GOS database in the analysis. This approach resulted in the identification of sequences from oceanic bacteria that seemed to be more closely related to the mitochondrial progenitor than previously recognized bacteria. In addition, we show that the SAR11 clade is not a sister-clade to the Rickettsiales-mitochondria clade, as was suggested by previous studies.

## Results

### Marker Gene Selection for Inferring the Phylogenetic Affiliation of Mitochondria and Alphaproteobacteria

**Figure 1:**
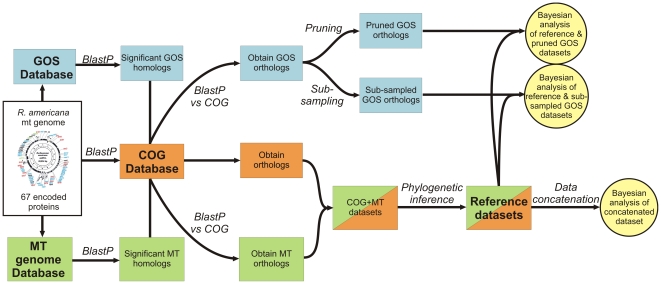
Schematic overview of data selection procedure. The flow scheme depicted here displays which datasets have been used and how they were analysed (for more details, see Materials and Methods). Three different datasets have been used, being the COG database (taken from STRING [40]; “COG”, orange shading), the GOS database (“GOS”, blue shading), and a local database of proteins that are encoded by mitochondrial genomes (‘MT’, green shading). First, homologs were retrieved for each of the 67 proteins encoded by the *R. americana* mitochondrial genome for each of these datasets using BlastP searches. Next, paralogs and distant homologs were removed from the retrieved GOS and MT hits by performing BlastP searches against the COG database and using stringent cut-off filters. Since the amounts of retrieved GOS homologs was too high for Bayesian analyses, two strategies were used for down-sampling: One approach involved a pruning step in which the amount of GOS homologs was reduced while reducing the phylogenetic diversity, another approach involved the targeted sub-sampling of GOS sequences that were placed as a neighbour to the mitochondrial clade in a jack-knifing screen (see Material and Methods for details). Then, the MT and COG datasets were combined and subjected to phylogenetic analysis (PhyML), selecting only those proteins whose evolutionary history was evolutionary coherent (i.e. Alphaproteobacteria formed one clade, and mitochondria formed one clade). The resulting protein datasets are referred to as the ‘reference datasets’. The reference datasets were used for three independent analyses: (i) Proteins of the reference dataset were concatenated and subjected to Bayesian analysis; Proteins of the reference dataset were either combined with the pruned (ii) or sub-sampled (iii) GOS datasets, followed by Bayesian analysis.

The starting point for our analysis was the mitochondrial proteome of the freshwater protist *Reclinomonas americana*, defined as the protein sequences that are encoded by its mitochondrial genome [21] (See Figure 1 for overview). First a reference dataset was composed for each protein of this proteome by extracting orthologous protein sequences from a set of 28 alphaproteobacterial genomes and up to 18 mitochondrial genomes (Table S1). To check for phylogenetic coherence in the reference data sets and to exclude protein datasets in which phylogenetic signals were potentially obscured by horizontal gene transfer events, multiple sequence alignments of the individual protein sequences in the reference datasets were generated and phylogenetic trees were inferred using PhyML [22]. Only those datasets in which the mitochondrial sequences formed a monophyletic group and in which the Alphaproteobacteria formed a monophyletic group were retained.

Ten datasets that fulfilled these criteria were selected for a more detailed phylogenetic analysis using Bayesian methods. These data sets included an essential enzyme of the citric acid cycle (SDH2), subunits of the ATP synthase complex (ATP1, ATP3) and components of each of the three energy-coupling sites of the respiratory chain: (i) the NADH dehydrogenase complex (NAD7, NAD8), (ii) the cytochrome *bc*1 complex (COB) and (iii) the cytochrome oxidase complex (COX1, COX2, COX3). For comparisons with bacteria without systems for oxidative phosphorylation, we also included ribosomal protein S2 (RPS2). These genes are present in most alphaproteobacterial genomes, although COX1, COX2 and COX3 have been lost independently from *Rhodospirillilum rubrum*, *Gluconobacter oxydans*, *Zymononas mobilis* and Bartonellaceae species. However, for each of the major alphaproteobacterial orders, a homolog was available for each of the 10 selected reference datasets, making the datasets comparable.

The subsequent Bayesian inference showed that of these 10 reference datasets, the COX1, COB and NAD7 proteins provided the highest support for the divergences of the deeper nodes and grouped the taxa into the six major alphaproteobacterial orders (Figures S1, S2, S3). To examine the influence of sequence heterogeneity among lineages [23]–[34] on our different protein data sets, we calculated the frequencies of amino acids coded solely by AT- and GC-codons in these species. Whereas a control dataset consisting of ribosomal proteins showed large variations in amino acid composition patterns among species, suggestive of mutational biases (Figure S4), the COX1, COB and NAD7 proteins were robust to such biases, displaying only a few percent differences in amino acid frequencies between the most AT- and GC-rich lineages (Table S2). Hence, the latter observation suggests that phylogenies based on these sequences are likely to be less influenced by base composition biases. In conclusion, the monophyly of mitochondria, the resolution of the deeper nodes into six major alphaproteobacterial orders and the lack of base composition biases suggest that COX1, COB and NAD7 are particularly suitable for phylogenetic analyses of mitochondrial origins.

### Extracting GOS Sequences for Respiratory Proteins

In order to exploit the taxonomic diversity in the GOS dataset for a study of the evolutionary relationship of mitochondria with bacteria from the ocean upper surface waters, we extracted full-length GOS sequences that produced significant best hits (BlastP, E-value<1*10^−10^ and >50% HSP overlap to the query protein) to the *R. americana* mitochondrial protein sequences through a series of subsequent filtering steps (Figure 1, see Material and Methods for details). The number of retrieved GOS sequences per protein differed markedly, ranging from several hundred to a few thousand sequences (Table S3). The observed differences in abundance are mainly the result of differences in gene length as well as anticipated differences in phylogenetic distribution patterns among different bacterial groups. For example, the short, universal RPS2 genes are much more abundant in the GOS dataset than COX1 genes, which are much longer and have a limited phylogenetic distribution.

**Figure 2:**
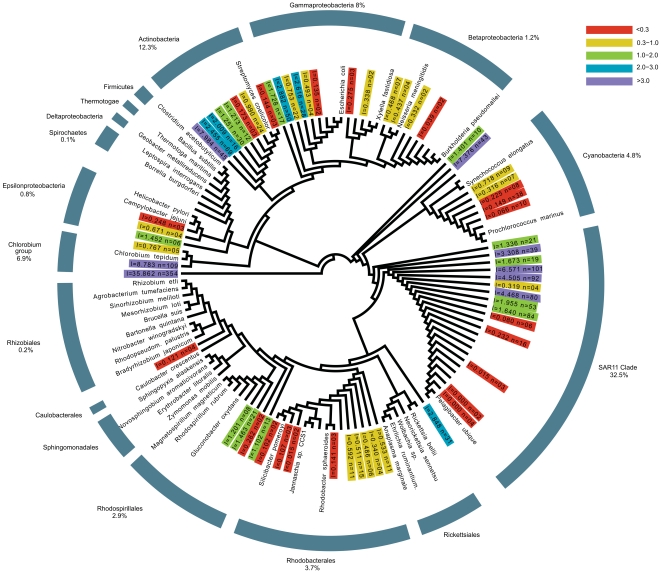
The taxonomic sampling of Alphaproteobacteria is enhanced by the addition of GOS sequences. A phylogenetic analysis of 1641 GOS RPS2 homologs reveals that the taxonomic sampling is increased for most major bacterial clades, and specifically for the Alphaproteobacteria. The vast majority (82.5%) of the extracted RPS2 homologs that are placed in the Alphaproteobacteria is associated with the SAR11 clade, which includes *Ca.* Pelagibacter ubique. Rhodobacterales, Rhodospirillales, Rhizobiales and Rickettsiales are associated with 9.3, 7.4, 0.6 and 0.2% of the GOS sequences, respectively. The sequence data covered by the GOS dataset greatly increases the previous sampling of the SAR11-clade, as well as of other oceanic bacteria that lack sequenced representatives, thereby representing a rich source of sequences useful for examining the placement of mitochondria in relation to bacteria adapted to the upper surface waters of the oceans. For clarity, all GOS clades have been collapsed. Annotation of collapsed GOS clades is as follows: *l* and *n* represent the total branch length and the number of GOS sequences within collapsed GOS clades, respectively, and shading is according to the total branch length of the clade as indicated.

To get a rough estimate of the diversity of the retrieved sequences in relation to previous analyses of the diversity in the GOS metagenome data set, neighbour-joining trees including all sequences extracted from the GOS database were produced. As expected, for all genes, the dominant fraction of extracted sequences was affiliated with the SAR11 clade. For example, of the alphaproteobacterial RPS2 homologs extracted in our study, the vast majority, 82.5%, were tentatively associated with this clade (Figure 2). Other main alphaproteobacterial clades were less well represented, with Rhodobacterales, Rhodospirillales, Rhizobiales and Rickettsiales containing only 9.3%, 7.4%, 0.6% and 0.2% of the alphaproteobacterial GOS sequences, respectively. None of the extracted RPS2 homologs were affiliated with Sphingomonodales or Caulobacterales.

The estimated diversity of the extracted GOS sequences is in good agreement with a recent survey of GOS sequence diversity as inferred by ribotyping [20], except that our estimated abundance of Rhodospirillales (7.4%) is significantly higher than reported in the previous study (1.2%) [20]. The observed discrepancy is probably caused by the fact that no reference species with a sequenced genome were available at the time of our analysis from the relatively abundant SAR116 cluster, which is distantly related to Rhodospirillales [35]. As a result, SAR116-derived RPS2 homologs present in the GOS data have most likely been accounted as Rhodospirillales sequences in our study. Consistently, the SAR116 clade represents 6.3% of the alphaproteobacterial ribotypes [20].

Altogether, the sequence data covered by the GOS dataset greatly increases the previous sampling of the SAR11-clade, as well as of other oceanic bacteria that lack sequenced representatives, thereby representing a rich source of sequences for examining the placement of mitochondria in relation to bacteria found in the upper surface waters of the oceans. To do so, we set out to determine the placement of the extracted GOS sequences relative to the recognized alphaproteobacterial and mitochondrial sequences in each of the COX1, COB and NAD7 protein trees. Given that Bayesian inferences of protein phylogenies are computationally demanding, we had to down-sample the amount of GOS sequences prior to performing such analyses. To achieve this, we used two different approaches: (i) sub-sampling the data while maintaining phylogenetic diversity (pruning), and (ii) pre-filtering the data for specific GOS homologs that grouped as potential mitochondrial neighbour, as detailed below.

### Inclusion of a Pruned Set of Genetically Diverse GOS Sequences

**Figure 3:**
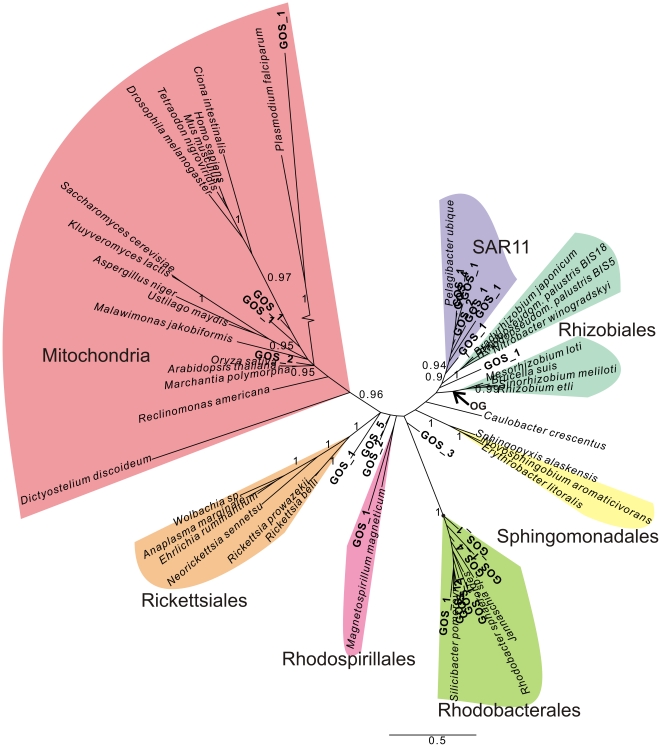
Phylogenetic analysis COX1 orthologs extracted from the GOS database. A phylogenetic tree is shown that is based on an alignment of COX1 protein sequences from the reference set of alphaproteobacterial and mitochondrial species supplemented with a pruned set of GOS sequences (shown in bold). The main alphaproteobacterial orders Rickettsiales, Rhodobacteriales, Rhodospirillales, Sphingomonadales, Caulobacteriales and Rhizobiales are indicated in coloured shading. Note that some GOS sequences are placed close to or at the root of the Rickettsiales clade and that the SAR11 clade encompassing *Ca.* Pelagibacter ubique is unrelated to the mitochondrial lineage. The tentative placement of the outgroup (OG) is indicated with an arrow. Phylogenies were produced using Bayesian methods with the CAT model. Numbers at nodes denote posterior probability values. Numbers associated with GOS clades denote the number of GOS sequences here represented as a single terminal node.

**Figure 4:**
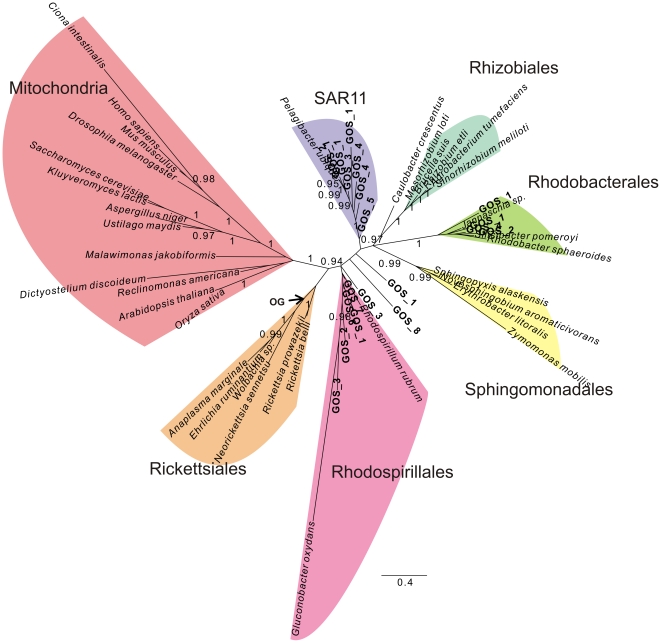
Phylogenetic analysis COB orthologs extracted from the GOS database. A phylogenetic tree is shown that is based on an alignment of COB protein sequences from the reference set of alphaproteobacterial and mitochondrial species supplemented with a pruned set of GOS sequences (shown in bold). The main alphaproteobacterial orders Rickettsiales, Rhodobacteriales, Rhodospirillales, Sphingomonadales, Caulobacteriales and Rhizobiales are indicated in coloured shading. Note that the SAR11 clade encompassing *Ca.* Pelagibacter ubique is unrelated to the mitochondrial lineage. The tentative placement of the outgroup (OG) is indicated with an arrow. Phylogenies were produced using Bayesian methods with the CAT model. Numbers at nodes denote posterior probability values. Numbers associated with GOS clades denote the number of GOS sequences here represented as a single terminal node.

**Figure 5:**
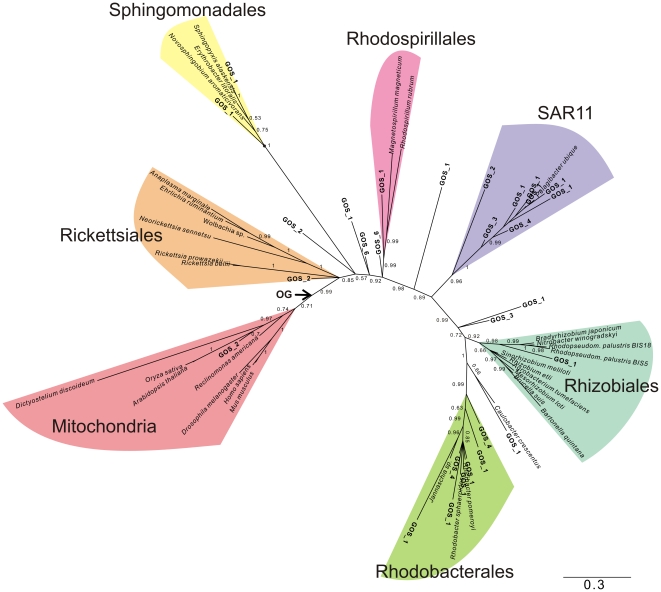
Phylogenetic analysis NAD7 orthologs extracted from the GOS database. A phylogenetic inference based on an alignment of NAD7 protein sequences from the reference set of alphaproteobacterial and mitochondrial species supplemented with a pruned set of GOS sequences (shown in bold). The main alphaproteobacterial orders Rickettsiales, Rhodobacteriales, Rhodospirillales, Sphingomonadales, Caulobacteriales and Rhizobiales are indicated. Note that some GOS sequences are placed at the root of the Rickettsiales clade and that the SAR11 clade encompassing *Ca.* Pelagibacter ubique is unrelated to the mitochondrial lineage. The tentative placement of the outgroup (OG) is indicated with an arrow. Phylogenies were produced using Bayesian methods with the CAT model. Numbers at nodes denote posterior-probability values. Numbers associated with GOS clades denote the number of GOS sequences here represented as a single terminal node.

In order to obtain manageable amounts of COX1, COB and NAD7 homologs from the GOS database, a pruning step was incorporated such that the number of sequences decreased while the phylogenetic diversity within the dataset was maintained (See Material and Methods for details). This pruning procedure resulted in the addition of 30–50 GOS sequences per protein dataset. Despite the pruning step that eliminated much of the genetic redundancy, a large majority of the GOS sequences still clustered with *Ca.* Pelagibacter ubique with posterior probability values (pp) in the range of 0.90–0.97 (Figures 3, 4, 5). Another set of GOS sequences clustered within the Rhodobacterales (pp = 0.99–1), which contains the oceanic bacteria *Silicibacter pomeroyii* and *Jannaschia* sp. CCS1. A third set of GOS sequences were placed within the Rhodospirilalles (pp = 0.94–0.99). Theoretically, the GOS database should not contain any sequences of eukaryotic origins since the pore size of the filters that were utilized during the sampling procedures was specifically aimed at recovering bacteria. Yet, we observed that a number of GOS sequences grouped internally with the mitochondrial lineage of the COX1 tree with high support (Figure 3). Most likely, these sequences are derived from photosynthetic picoeukaryotes related to green alga or to marine alveolates [36].

Interestingly, some COX1 and NAD7 sequences were identified in the GOS dataset that were placed close to or at the root of the Rickettsiales and in relatively close proximity of the mitochondrial clade (Figure 3 and 5). Encouraged by these results, we decided to use a targeted approach to identify more GOS sequences that could belong to these oceanic clades.

### Systematic Searches for Mitochondrial Neighbours in the GOS Data

To systematically search for sequences that are more closely related to the mitochondrial clade than any of the alphaproteobacterial reference species, we designed a taxon jack-knifing procedure in which GOS sequences were extracted that clustered in the vicinity of the mitochondrial clade in phylogenetic analyses. To this end, random samples of 100 GOS sequences were extracted for each of the selected proteins and added to the sequences in the reference data set. These combined datasets were used to construct phylogenetic trees using RAxML under the CAT [WAG] model. Subsequently, sequences affiliated with the mitochondrial clade were selected for further analysis using Bayesian methods. This procedure was repeated 100 times in order to ensure that all sequences in the dataset were sampled (the estimated un-sampled fraction for the largest dataset ∼6.7*10^−5^). A random sampling approach was used to avoid sampling biases.

**Figure 6:**
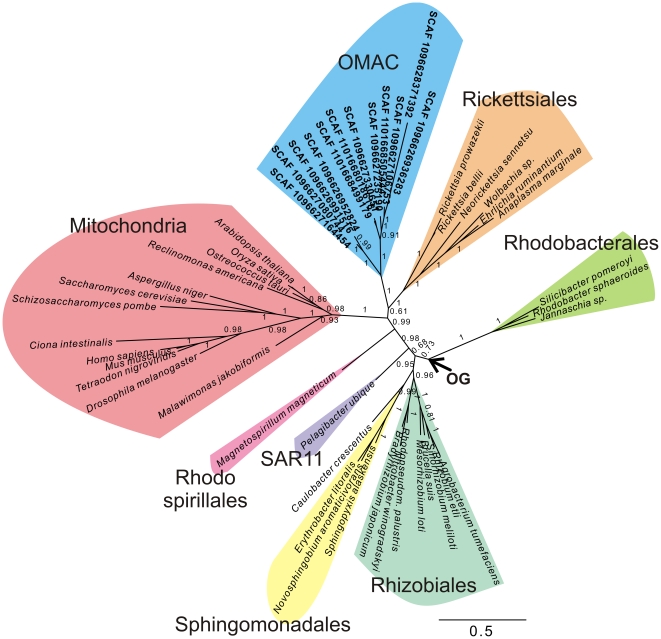
Deeply diverging alphaproteobacterial GOS sequences in a COX1–COX2 protein tree. A systematic search for GOS sequences was performed to identify alphaproteobacterial clades that could potentially represent the closest free-living ancestor of the mitochondria. The Bayesian phylogenetic analysis of concatenated COX1–COX2 sequences encoded by GOS scaffolds revealed a deeply diverging clade of GOS sequences that are associated with the Rickettsiales (pp = 0.99). The tentative placement of the outgroup (OG) is indicated with an arrow. Phylogenies were produced using Bayesian methods with the CAT model. Numbers at nodes denote posterior-probability values.

Several of the resulting Bayesian single protein trees indicated the presence of deeply diverging GOS sequences, but the topology was often poorly resolved (not shown). In an attempt to improve the resolution, we selected in a second step GOS sequences situated on scaffolds (i.e. continuous stretches of sequences obtained from the assembly procedure of GOS data) with more than one gene per scaffold. A Bayesian analysis of the concatenated COX1 and COX2 proteins revealed a clade comprising GOS sequences, the Rickettsiales and mitochondria with a posterior probability of 0.99 (Figure 6), as also shown by a network analysis (Figure S5). These findings allude to the existence of a hitherto undetected clade of Alphaproteobacteria that may represent free-living relatives of the Rickettsiales and, by inference, extant relatives of the mitochondrial progenitor.

We propose to refer to this clade as OMAC, after Oceanic Mitochondrial Affiliate Clade. Based on low fraction of COX1 sequences that were retrieved in the GOS dataset that can be reliably attributed to this clade (12 out of 566; 2.1%), OMAC does not seem to be particularly abundant in the ocean. Under the assumption that Alphaproteobacteria comprise ∼32.5% of all microbial cells (see Figure 2, based on phylogenetic distribution of RPS2 sequences), the relative overall abundance of OMAC is estimated to be less than 1 percent (0.84%) of all cells. Such sequences were identified in both open ocean and coastal waters, and in both temperate and tropical waters (Table 1). These bacteria seemed not to be associated with any particular habitat (Table 1) although 4 out of 12 OMAC COX1 sequences were derived from three Sargasso Sea sampling stations. However, given the low numbers of sequences that were identified, the differences in sequencing depth between the different GOS sampling sites, and the existence of temporal variation of microbial cell abundances, any inference of cell abundance should be taken with extreme caution.

**Table 1:** Geographic location of OMAC scaffolds encoding COX1–COX2 sequences that were identified in the GOS dataset.

OMAC Scaffold id	GOS ID	Habitat type	Sample location	Coordinates
JCVI_SCAF_1096627235190	GS000b	Open ocean	Sargasso Sea, Station 13 and 11	31°32′10n; 63°35′70w
JCVI_SCAF_1096626936283	GS000d	Open ocean	Sargasso Sea, Station 13	31°32′6n; 63°35′42w
JCVI_SCAF_1096626952824	GS000d	Open ocean	Sargasso Sea, Station 13	31°32′6n; 63°35′42w
JCVI_SCAF_1096628371392	GS001b	Open ocean	Sargasso Sea, Hydrostation S	32°10′00n; 64°30′00w
JCVI_SCAF_1101668018618	GS002	Coastal	Gulf of Maine	42°30′11n; 67°14′24w
JCVI_SCAF_1096626991516	GS018	Open ocean	Rosario Bank	18°2′12n; 83°47′5w
JCVI_SCAF_1096627080744	GS027	Coastal	Devil's Crown, Floreana Island	1°12′58s; 90°25′22w
JCVI_SCAF_1096627330651	GS029	Coastal	North James Bay, Santigo Island	1°12′58s; 90°25′22w
JCVI_SCAF_1096627106753	GS030	Warm seep	Warm seep, Roca Redonda	0°12′0s; 90°50′7w
JCVI_SCAF_1101668499179	GS031	Coastal upwelling	Upwelling, Fernandina Island	0°18′4s; 91°39′6w
JCVI_SCAF_1101668505444	GS031	Coastal upwelling	Upwelling, Fernandina Island	0°18′4s; 91°39′6w
JCVI_SCAF_1096627164454	GS036	Coastal	Cabo Marshall, Isabella Island	0°35′38s; 91°4′10w

### SAR11 is not closely related to the Rickettsiales/mitochondria clade

**Figure 7:**
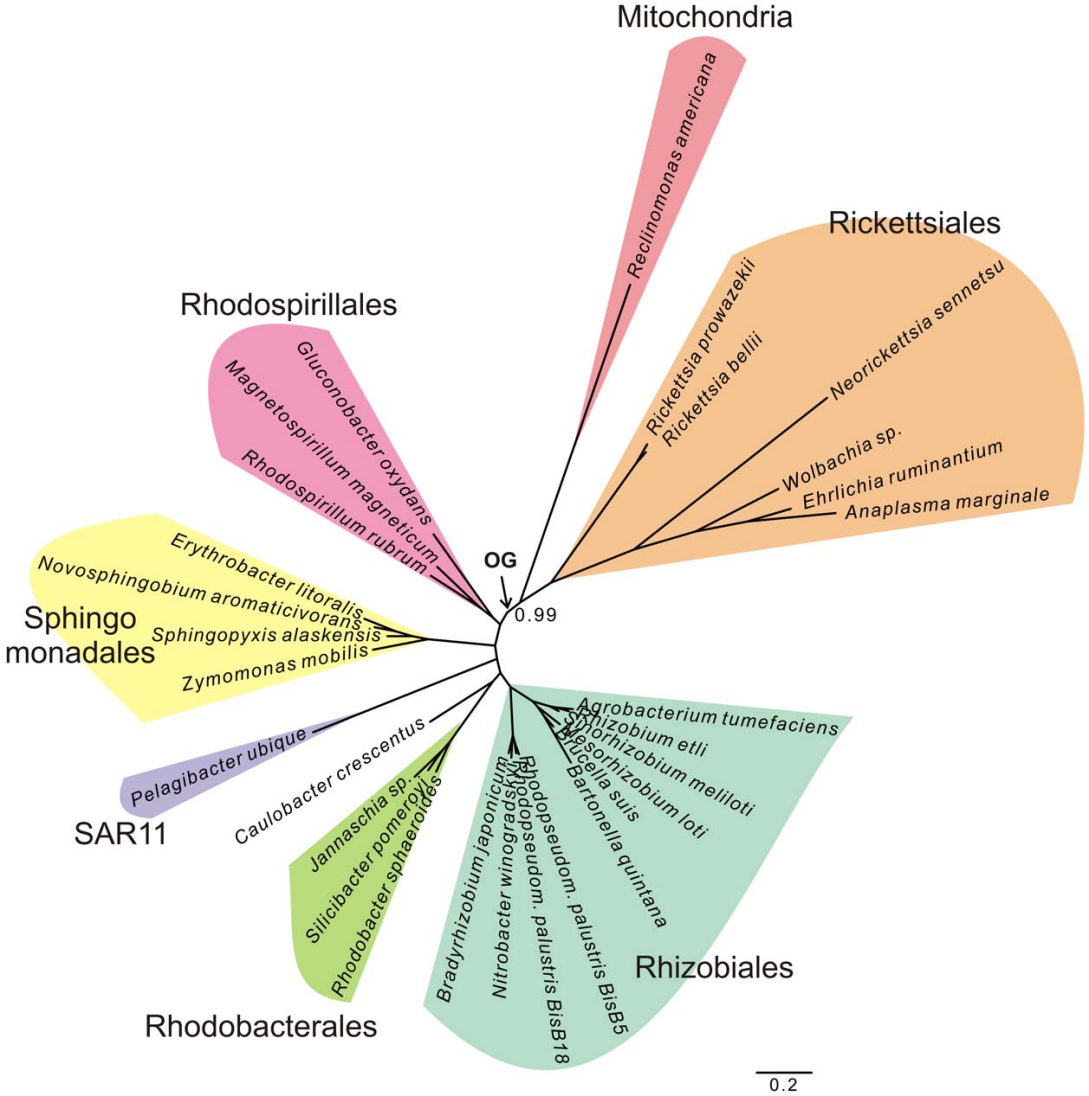
*Ca.* Pelagibacter ubique is placed with free-living Alphaproteobacteria in a phylogenetic tree inferred from concatenated protein sequences. A phylogenetic inference of the alphaproteobacterial tree topology based on a concatenated alignment of 42 mitochondrially encoded proteins reveals a well-supported alphaproteobacterial phylogeny, in which the SAR11 clade, here represented by *Ca.* Pelagibacter ubique is placed among the free-living alphaproteobacteria rather than with the Rickettsiales. The tentative placement of the outgroup (OG) is indicated with an arrow. Phylogenies were produced using Bayesian methods with the CAT model. Posterior-probability values of 1 are not shown.

As part of the Bayesian analyses of datasets that included genetically diverse sets of GOS sequences (Figures 3, 4, 5), we noticed that the SAR11 clade, comprising *Ca.* Pelagibacter ubique and affiliated GOS sequences, showed a tendency to cluster with free-living Alphaproteobacteria, rather than with the Rickettsiales/mitochondria clade, as has been suggested in previous studies [15], [37]. To investigate this further, we inferred a phylogeny of a concatenated dataset comprising 42 proteins encoded by the *R. americana* mitochondrial genome and their alphaproteobacterial orthologs (Table S4). Using Bayesian methods under the CAT-model [30], a well-supported tree topology was obtained that resolved all major alphaproteobacterial orders (Figure 7). Importantly, *Ca.* Pelagibacter ubique, the sole representative of the SAR11 clade was embedded within the group of free-living Alphaproteobacteria (Rhodobacteriales, Rhodospirillales, Sphingomonadales, Caulobacteriales and Rhizobiales) with high support in this tree, whereas *R. americana* was placed as a sister lineage to the Rickettsiales (Figure 7).

## Discussion

The current study was motivated by a recent phylogenetic survey that identified the closest free-living sister clade of mitochondria and the Rickettsiales to be SAR11, [15], a clade that dominates the ocean surface waters in terms of abundance [18]. Our aim was to explore the recently published ocean surface metagenome [19] to search for genes in the oceans from free-living bacteria that might be related to the mitochondrial progenitor. As such, this study describes the first systematic attempt to infer the evolutionary relationships of mitochondrial proteins involved in oxidative phosphorylation to metagenomic sequences recovered from the oceans.

In the present study, we have identified a group of bacteria in the oceans that is more closely related to mitochondria than the SAR11 clade or the Rhodospirillales, the only two groups in the tree that contain cultivated species from the oceans. The new group, here referred to as the OMAC clade, is represented by GOS sequences in relatively low abundance based on the fact that we could attribute only 12 COX1 sequences in the GOS dataset to this group (less than 1% of the cell population of the ocean surface waters). Ribotyping surveys of GOS data have revealed the presence of Alphaproteobacteria that are currently unclassified or belong to uncultured clades, such as for example the SAR116 cluster [20]. Novel sequences from poorly characterized alphaproteobacterial species are therefore to be expected.

Apart from identification of the OMAC clade, our study associates *Ca.* Pelagibacter ubique and the SAR11 clade with the group of free-living Alphaproteobacteria rather than with mitochondria and the Rickettsiales in the phylogenetic analyses of both concatenated and single protein datasets. These results corroborate the outcome of an independent study performed in our group that also supported a clustering of *Ca.* Pelagibacter ubique with marine and soil bacteria [38]. The genomes of *Ca.* Pelagibacter ubique and members of the Rickettsiales are AT-rich, whereas most other alphaproteobacterial genomes are GC-rich. Phylogenies inferred from sites in the alignment that were strongly affected by these biases supported a clustering of Ca. Pelagibacter ubique with the Rickettsiales, whereas sites less affected by such compositional heterogeneity clustered Ca. Pelagibacter ubique with free-living Alphaproteobacteria [38]. Thus, the clustering of the SAR11 clade with the Rickettsiales observed previously is likely to be an artefact of the strong AT/GC bias in the dataset. Since COX1, COB and NAD7 are three of the most highly conserved mitochondrial proteins and thereby less sensitive to these biases, we suggest that the separation of the SAR11 clade from mitochondria and the Rickettsiales observed in our phylogenies represent the underlying evolutionary divergence pattern. It is reassuring that the position of the Rickettsiales with the same low genomic G + C content as *Ca.* Pelagibacter ubique was stable in all our phylogenies.

Given that the process of oxygenation probably started in the ocean surface waters, it seems reasonable to assume that the systems for aerobic respiration evolved in the oceanic surface waters as well. However, much of the genetic diversity present in the ancestral oceans may since have been eliminated. Hence even if mitochondria originated in the oceans, its most closely related free-living bacterial relative may not necessarily be highly abundant in the modern oceans. Furthermore, periods of rapid evolutionary change along with different selective constraints on respiratory chain proteins in the eukaryotic cells and in free-living bacterial cells in the oceans may make it very difficult to trace these relationships using the currently available methods and tools. Despite these limitations it is encouraging to see that novel mitochondrial sequence relatives can be identified in metagenomic data sets. Whole-genome sequence analyses of these and other close relatives may thus help resolve some of the many questions concerning the origin and evolution of mitochondria.

### Concluding remarks

The findings presented in the current study underscore that future research aiming at the identification and culturing of bacterial lineages related to the mitochondrial progenitor should regard the exploration of marine environments such as the ocean surface waters as a priority goal. Additionally, models on the origin and evolution of the eukaryotic cell and its organelles now need to be re-examined in light of the full genetic diversity of micro-organisms that is being uncovered by metagenome sequence data. Analyses combining the increasing volumes of sequence data with computationally intense evolutionary methods will require the development of new frameworks in bio-informatics. The recent development of improved analytical methods and the rapid increase of processing power give good hope that these fundamental biological questions can be further resolved.

## Materials and Methods

### Delineation and Selection of Reference Datasets

First, each of the 67 proteins encoded by the *Reclinomonas americana* mitochondrial genome were assigned to an orthologous group of proteins by performing BlastP searches [39] against an updated COG database [40], comprising proteomes of a balanced selection of species, including species from all major bacterial, archaeal and eukaryotic divisions. *R. americana* proteins were assigned to an orthologous group if a significant, best hit (E-value<1*10^−10^, HSP (high-scoring segment pair) overlap >50%) was observed against a protein of this orthologous group. To remove any redundancy, only the best hit against a *R. americana* protein was retained in cases where an orthologous group contained multiple proteins from the same species. Given the central position of the Alphaproteobacteria in the current analysis, we included all available alphaproteobacterial genomes represented in the STRING database (Table S1).

In order to increase the phylogenetic coverage of mitochondrial proteins, and to compensate for those cases in which mitochondrially encoded proteins were omitted from the eukaryotic proteomes covered in the STRING database, we included mitochondrially encoded proteins for a selected number of species in our analysis (Table S1). The selected mitochondrial genomes were searched for potential orthologs for each of the 67 proteins encoded by the *R. americana* mitochondrial genome by BlastP analysis. Significant hits (E-value<1*10^−10^, HSP-overlap>50%) were added to the respective COGs. For those species where a mitochondrial protein was already represented in a given orthologous group, only one copy was retained. The respective orthologous groups, supplemented with the selected mitochondrial sequences are referred to as the ‘reference datasets’ throughout the manuscript.

### Retrieval and Filtering of GOS Sequences

Selection of GOS sequences was initiated by performing BlastP analyses [39] against the CAMERA protein database [41] using the mitochondrially encoded *R. americana* proteins as a query. In order to filter out potential paralogs and distant homologs, each GOS sequence thus identified (E-value<1*10^−10^, HSP-overlap>50%) was then used as a query in a BlastP search against the updated COG database [40]. The GOS sequence was retained for further analysis only if the top hit (E-value<1*10^−10^, HSP-overlap>70%) was a member of the same orthologous group as the *R. americana* query protein. In order to warrant sufficient diversity covered in the environmental datasets, only datasets for which more then 500 GOS sequences were retrieved in the first BlastP search were considered.

### Pruning the GOS Dataset

To select a manageable number of GOS sequences to include in the phylogenetic analyses while trying to maintain as high a diversity as possible among the included environmental sequence, sequences were selected to maximize the phylogenetic diversity [42] for a growing set using a greedy algorithm [43] as implemented in the in-house software MrTwig [44]. A set of 150 sequences was determined to be a feasible set while still comprising representatives from all major (tentative) clades observed in the full selection of GOS sequences.

### Phylogenetic Inference of Concatenated Gene Trees

A concatenated dataset of 42 protein sequences (Table S4) was assembled as follows: For each of the 42 proteins, sequence alignments were constructed using Kalign 2.03 [45] that, apart from the *Reclinomonas* sequence, included up to 28 alphaproteobacterial orthologs. The outgroup consisted of homologous protein sequences from *Escherichia coli*, *Pseudomonas aeruginosa*, *Helicobacter pylori* and *Campylobacter jejuni* when available. The protein alignment (Dataset S1) was cleaned with Gblocks [46] using default settings. Subsequently, a concatenated dataset was constructed which was used for phylogenetic inference by running Bayesian analyses using PhyloBayes 3.2c under the CAT+G+I model [30]. In order to prevent obtaining phylogenies that are a result of chains that got stuck in local optima, several chains were analysed and compared.

### Phylogenetic Inference of Single Protein Trees

To check for taxonomic consistency multiple sequence alignments of the protein sequences in the reference datasets were created using Kalign 2.03 [45] and phylogenetic trees were constructed using PhyML [22]. We used PhyloBayes 3.2c to run Bayesian analyses using the CAT+G+I model for the selected set of single protein trees that included 28 alphaproteobacterial orthologs. The outgroup consisted of homologous protein sequences from *Ralstonia solanacearum* and *Burkholderia pseudomallei* [30]. Two chains were run in all cases and convergence was checked by plotting the parameters and discarding 25% of all trees after a stable state was reached, after an initial step in which output from the bpcomp program indicated a max-diff value of no more than 0.3. The remaining trees were summarized after removal of burn-in, both as majority rule consensus trees and as consensus networks using SplitsTree 4 [47], [48]. For the consensus network, a subsample of *c.* 500 trees from the trees after burn-in was used for each gene, and consensus networks were made using a threshold value of 0.25.

### Identification of Potential Mitochondrial Neighbour Sequences from the GOS Dataset

We used a step-wise approach to search for GOS sequences forming sister-taxons to the mitochondria, or that are situated within the mitochondrial clade. First, we performed a jack-knife analysis using the full set of extracted GOS sequences together with the reference ortholog groups for each gene. Using an in-house Perl script, random samples of 100 homologous sequences were drawn with replacement from the GOS data and aligned with all the sequences in the reference data set. For each gene and sample size, 100 replicates were generated with RAxML and the CAT-model of protein substitution. GOS sequences associated with the mitochondrial clade were automatically extracted using a script developed in-house. To improve the resolution in the subsequent phylogenetic analyses, we used a second filtering step to select GOS sequences situated on a scaffold that comprises more than one gene. This step assured that the final alignment consisted of two, or in a few cases three, concatenated genes.

Finally, we aligned the concatenated GOS and reference sequence datasets, and performed Bayesian phylogenetic inference using Phylobayes and the CAT-model of protein substitution. In the COX1–COX2 protein tree the outgroup consisted of concatenated protein sequences from *R. solanacearum*, *B. pseudomallei*, *P. aeruginosa*, *Mycobacterium tuberculosis*, *Corynebacterium glutamicum*, *Streptomyces coelicor*, *Xyellla fastidiosa*, *Bacillus subtilis*, *Geobacter metallireducens*, *Desulfovibrio vulgaris*, *Leptospira interrogans* and *Anaeromyxobacter dehalogenans*.

## Supporting Information

Figure S1 **Placement of mitochondria in a protein tree inferred from an alignment of COX1 protein sequences.** A phylogenetic inference based on an alignment of COX1 protein sequences from the reference set of alphaproteobacterial and mitochondrial species. The main alphaproteobacterial orders Rickettsiales, Rhodobacteriales, Rhodospirillales, Sphingomonadales, Caulobacteriales and Rhizobiales are indicated. Note that the SAR11 clade encompassing *Ca.* Pelagibacter ubique is unrelated to the mitochondrial lineage. The tentative placement of the outgroup (OG) is indicated with an arrow. Phylogenies were produced using Bayesian methods with the CAT model. Numbers at nodes denote posterior-probability values.
(EPS)

Figure S2 **Placement of mitochondria in a protein tree inferred from an alignment of COB protein sequences.** A phylogenetic inference based on an alignment of COB protein sequences from the reference set of alphaproteobacterial and mitochondrial species. The main alphaproteobacterial orders Rickettsiales, Rhodobacteriales, Rhodospirillales, Sphingomonadales, Caulobacteriales and Rhizobiales are indicated. Note that the SAR11 clade encompassing *Ca.* Pelagibacter ubique is unrelated to the mitochondrial lineage. The tentative placement of the outgroup (OG) is indicated with an arrow. Phylogenies were produced using Bayesian methods with the CAT model. Numbers at nodes denote posterior-probability values.
(EPS)

Figure S3 **Placement of mitochondria in a protein tree inferred from an alignment of NAD7 protein sequences.** A phylogenetic inference based on an alignment of NAD7 protein sequences from the reference set of alphaproteobacterial and mitochondrial species. The main alphaproteobacterial orders Rickettsiales, Rhodobacteriales, Rhodospirillales, Sphingomonadales, Caulobacteriales and Rhizobiales are indicated. Note that the SAR11 clade encompassing *Ca.* Pelagibacter ubique is unrelated to the mitochondrial lineage. The tentative placement of the outgroup (OG) is indicated with an arrow. Phylogenies were produced using Bayesian methods with the CAT model. Numbers at nodes denote posterior-probability values.
(EPS)

Figure S4 **Biased amino acid composition patterns.** Frequencies of amino acids encoded exclusively by AT (A) and GC (B) codons for whole proteomes and for ribosomal proteins (RP) encoded by the genomes of *Ca.* Pelagibacter ubique, *Rickettsia prowazekii* and *Caulobacter crescentus*.
(EPS)

Figure S5 **Network analysis of a concatenated dataset of COX1 and COX2 protein sequences including sequences from the OMAC group.** The network analysis revealed a deeply diverging clade of GOS sequences that are associated with the Rickettsiales. The main alphaproteobacterial orders Rickettsiales, Rhodobacteriales, Rhodospirillales, Sphingomonadales, Caulobacteriales and Rhizobiales are indicated.
(EPS)

Table S1 Species included in the analysis.
(XLS)

Table S2 Frequencies of amino acids coded by AT- and GC-codons of COX1, COB and NAD7 proteins encoded by alphaproteobacterial genomes.
(XLS)

Table S3 Results of BlastP searches against the GOS metagenome database using mitochondrial proteins from *R. americana* proteins as query (E<1*10^−10^, HSP overlap of query protein >50% of the total length). Each hit was subsequently used as a query against the COG database and only those sequences that had a best hit in the same orthologous group as the *R. americana* seed protein was retained (E<1*10^−10^, HSP overlap>70%).
(XLS)

Table S4 Mitochondrial proteins used in the concatenated alignment used to produce the phylogeny shown in Figure 7.
(XLS)

Dataset S1 Concatenated alignment of 42 mitochondrial proteins from *Reclinomonas americana* and 28 alphaproteobacterial orthologs. Outgroup sequences from *Escherichia coli*, *Pseudomonas aeruginosa*, *Helicobacter pylori* and *Campylobacter jejuni* were included when available.
(AFA)
